# Translating benefits of exercise on depression for youth with autism spectrum disorder and neurodevelopmental disorders

**Published:** 2017-03-30

**Authors:** Eve Spratt*, Mary Ashley Mercer, Allie Grimes, Carrie Papa, Jessa Norton, Alexandra Serpe, Martina Mueller, Mark Eckert, Katie Harris, Lee Blackmon, James Durant, Janis Newton

**Affiliations:** Division of Developmental-Behavioral Pediatrics, Medical University of South Carolina, 135 Rutledge Avenue, MSC 561, Charleston, SC, USA

## Abstract

Young adults with Autism Spectrum Disorder (ASD) are at increased risk of poor health and depressive symptoms due to difficulty with communication, limited interests, sensory deficits, and sedentary lifestyles. The Piece It Together program was developed to provide socialization and wellness goals for teens and young adults with ASD and other mild neurocognitive deficits. The curriculum includes strength and endurance training, nutrition education, and stress reduction techniques to promote healthy lifestyle choices. Twelve participants, aged 15–27, attended 90-minute classes at the MUSC Wellness Center twice a week for six weeks and were encouraged to increase their activity level every day. The Patient Health Questionnaire (PHQ-9) was administered to each participant at the first and last class. Results showed significantly decreased PHQ-9 depression scores at the end of the program, from mild (7.69) to minimal depression (3.42) (p=0.000063). Overall, the activities completed in the Piece It Together program successfully unified this group to promote healthy lifestyle choices and resulted in improved mood.

## Introduction

Autism Spectrum Disorder (ASD) is an increasingly prevalent disorder and occurs in 1 in 68 children in the United States [1–5]. Young adults with ASD have an increased risk of poor health due to difficulty with communication, limited interests, sensory deficits, and sedentary lifestyles [1,3]. In addition, as many as 72% of children with ASD have a psychiatric co-morbidity [6]. The prevalence of depression in teens with ASD is approximately seven times higher than teens without ASD [7]. Encouraging the implementation of interventions that work to improve depression can be challenging for any population but even more difficult for youth with ASD due to traits associated with their disability. There have been few studies to explore the treatment of depression in youth with ASD; however, some intervention studies suggest a multi-modal approach including both psychosocial and psychopharmacological interventions [8]. There is an increasing awareness that physical activity is critically important for the advancement of overall wellness including the promotion of positive mental health. Exercise not only increases the levels of endorphins and vital neurotransmitters, such as serotonin, norepinephrine, and dopamine, but physical activity also builds confidence in the ability to cope with life stressors [9]. There is recognition that in some cases, exercise works as well as antidepressants [10]. Exercise regulates all of the same neurotransmitters targeted by antidepressants and exercise can be as good as medication [11]. Research has revealed that as little as 10 minutes of exercise can immediately improve vigor and mood in healthy subjects [12].

Teenagers with ASD or other neurodevelopmental disorders are far less likely to exercise or play team sports than are their typical peers [13]. Sedentary behavior has been linked to a 25% higher likelihood of being depressed when compared with non-sedentary people [14]. In a meta-analysis report, exercise was found as an effective intervention for managing depressive symptoms [15]. For youth with ASD, physical activity and aerobic exercise interventions have shown significant decreases in stereotypies, aggression, and off-task behavior as well as improvements in academic engagement and appropriate motor behavior following vigorous exercise [16].

Parents of children with ASD frequently report that the lack of developmentally appropriate programs and inherent sensory or psychological restrictions limit their child’s physical activity [3]. Given that opportunities for participation in leisure, recreation, or competitive fitness programs are very limited [17,18] Piece It Together (PIT) was developed and implemented as a summer day camp program to provide an environment where the implementation of physical activity could be successful for youth with special health care needs. The curriculum includes strength and endurance training, nutrition education, socialization, and stress reduction techniques to promote healthy lifestyle choices. Co-directors of the program include the fitness director of the Medical University of South Carolina Wellness Center and a physician specializing in pediatric medical and mental health.

## Methods

Seven males and five females, aged 15–27, attended 90-minute classes at the Medical University of South Carolina Wellness Center twice a week for six weeks over the summer. Eleven had ASD and one had a mild intellectual disability. The Patient Health Questionnaire (PHQ-9)^4^ was administered to each participant at the first and last class.

The general format of classes included 45 to 60 minutes of exercise, 15 to 30 minutes of stress reduction or mindfulness strategies, and 15 to 30 minutes of nutrition education totaling roughly 90-minutes per class. Physical activity was led by personal trainers and exercise physiologists and included spinning, water aerobics, walking/jogging, and circuit training. Participants were also encouraged to increase their activity level outside of the PIT group. Stress reduction and mindfulness activities consisted of yoga, meditation, positive selftalk, deep breathing, and stretching. As each participant had unique strengths and weaknesses, individualized nutrition, fitness, and socialization goals were established.

## Results

**Figure 1.**
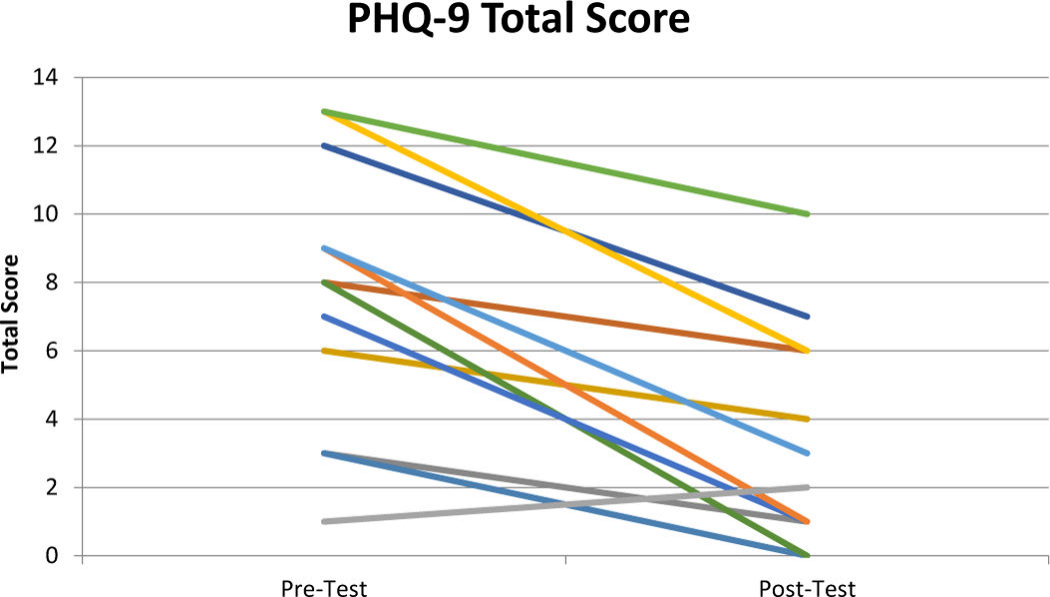
Comparison of average PHQ-9 scores from each of the twelve Piece It Together Participants at the first and last session of the six-week summer program.

Average PHQ-9 questionnaire scores decreased from mild (7.67) at the first class to minimal (3.42) at the last class (p=0.000063). See Figure 1 for comparison of individual PHQ-9 scores during the first and last classes of the six-week program. As the program promoted healthy lifestyle choices, additional results of participation in PIT included a modest improvement in self-efficacy related questions on the lifestyle questionnaire (p=0.032), an increase in fruit and vegetable consumption (p=0.038), and a statistically insignificant increase in skeletal muscle mass.

## Discussion

Physical activity is critically important for health promotion and disease prevention and the promotion of positive mental health, including improvements in mood and self-esteem. Exercise strengthens the cardiovascular system, boosts the immune system, and fortifies bones, but also fosters neuroplasticity and activates genes that protect cells against damage and disease.^9^ Additionally, routine exercise can spark biological changes that strengthen and condition the brain, similar to the way muscles grow with use and wither with inactivity.^9^ Remaining physically active is especially important in specific populations, such as those with ASD and other neurodevelopmental disorders due to increasingly sedentary lifestyles, which puts them at risk for poor overall health and depressive symptoms.

Participation in the PIT program not only helped to promote healthy lifestyle choices but also resulted in significantly improved mood amongst the participants. The participants’ improved mood supports the direct relationship between physical activity and mood. These results could further be generalized to broaden our understanding of how changing exercise habits can impact mood. Essentially, exercise is a mode of ‘active coping’ that gets one out of the territory of pessimism, fear, and retreat, and could be used as a targeted intervention in specific populations, such as those with ASD, to promote and encourage healthy, active lifestyles [9].

The PIT program helped to establish friendships, and many of the participants continue to socialize with each other outside of the program. Future studies are needed to determine maintenance and long-term health benefits, but these preliminary findings are encouraging.

## References

[1] Koenig K, De Los Reyes A, Cicchetti D, Scahill L, Klin A (2009) Group intervention to promote social skills in school-age children with pervasive developmental disorders: reconsidering efficacy. J Autism Dev Disord 39: 1163–1172. [Crossref] 19326199 10.1007/s10803-009-0728-1

[2] Strang JF, Kenworthy L, Daniolos P, Case L, Wills MC, (2012) Depression and Anxiety Symptoms in Children and Adolescents with Autism Spectrum Disorders without Intellectual Disability. Res Autism Spectr Disord 6: 406–412. [Crossref] 22615713 10.1016/j.rasd.2011.06.015 PMC3355529

[3] Yazdani S, Yee CT, Chung PJ (2013) Factors predicting physical activity among children with special needs. Prev Chronic Dis 10: E119 [Crossref] 23866163 10.5888/pcd10.120283 PMC3716337

[4] Kroenke K, Spitzer RL, Williams JB (2001) The PHQ-9: validity of a brief depression severity measure. J Gen Intern Med 16: 606–613. [Crossref] 11556941 10.1046/j.1525-1497.2001.016009606.x PMC1495268

[5] Christensen DL, Bilder DA, Zahorodny W, Pettygrove S, Durkin MS, (2016) Prevalence and Characteristics of Autism Spectrum Disorder Among Children Aged 8 Years - Autism and Developmental Disabilities Monitoring Network. J Dev Behav Pediatr 37: 1–8. [Crossref] 26651088 10.1097/DBP.0000000000000235

[6] Leyfer OT, Folstein SE, Bacalman S, Davis NO, Dinh E, (2006) Comorbid psychiatric disorders in children with autism: interview development and rates of disorders. J Autism Dev Disord 36: 849–861. [Crossref] 16845581 10.1007/s10803-006-0123-0

[7] Hedges S, White T, Smith L (2014) Depression in adolescents with ASD (Autism ata-Glance Brief) Chapel Hill: The University of North Carolina: Frank Porter Graham Child Development Institute, CSESA Development Team

[8] Ghaziuddin M, Ghaziuddin N, Greden J (2002) Depression in persons with autism implications for research and clinical care. J Autism Dev Disord 32: 299–306. [Crossref] 12199134 10.1023/a:1016330802348

[9] Ratey J, Hagerman E (2008) Spark: The Revolutionary New Science of Exercise and the Brain New York: Little, Brown and Company.

[10] Health Harvard. Exercise is an All-Natural Treatment to Fight Depression 8 2013; www.health.harvard.edu/mind-and-mood/exercise-is-an-all-natural-treatmentto-fight-depression. 27024674

[11] Blumenthal JA, Babyak MA, Moore KA, Craighead WE, Herman S, (1999) Effects of exercise training on older patients with major depression. Arch Intern Med 159: 2349–2356. [Crossref] 10547175 10.1001/archinte.159.19.2349

[12] Hansen CJ, Stevens LC, Coast JR (2001) Exercise duration and mood state: how much is enough to feel better? Health Psychol 20: 267–275. [Crossref] 11515738 10.1037//0278-6133.20.4.267

[13] Mangerud WL, Bjerkeset O, Lydersen S, Indredavik MS (2014) Physical activity in adolescents with psychiatric disorders and in the general population. Child Adolesc Psychiatry Ment Health 8: 2 [Crossref] 24450542 10.1186/1753-2000-8-2 PMC3914726

[14] Zhai L, Zhang Y, Zhang D (2015) Sedentary behaviour and the risk of depression: a meta-analysis. Br J Sports Med 49: 705–709. [Crossref] 25183627 10.1136/bjsports-2014-093613

[15] Kvam S, Kleppe CL, Nordhus IH, Hovland A (2016) Exercise as a treatment for depression: A meta-analysis. J Affect Disord 202: 67–86. [Crossref] 27253219 10.1016/j.jad.2016.03.063

[16] Lang R, Koegel LK, Ashbaugh K, Regester A, Ence W, (2010) Physical exercise and individuals with autism spectrum disorders: A systematic review. Res in Autism Spect Disord 4: 565–576.

[17] Murphy NA, Carbone PS, American Academy of Pediatrics Council on Children With D (2008) Promoting the participation of children with disabilities in sports, recreation, and physical activities. Pediatrics 121: 1057–1061. [Crossref] 18450913 10.1542/peds.2008-0566

[18] Newacheck PW, Strickland B, Shonkoff JP, Perrin JM, McPherson M, (1998) An epidemiologic profile of children with special health care needs. Pediatrics 102(1 Pt 1): 117–123. [Crossref] 9651423 10.1542/peds.102.1.117

